# A review of varietal change in roots, tubers and bananas: consumer preferences and other drivers of adoption and implications for breeding

**DOI:** 10.1111/ijfs.14684

**Published:** 2020-07-26

**Authors:** Graham Thiele, Dominique Dufour, Philippe Vernier, Robert O. M. Mwanga, Monica L. Parker, Elmar Schulte Geldermann, Béla Teeken, Tesfamicheal Wossen, Elisabetta Gotor, Enoch Kikulwe, Hale Tufan, Sophie Sinelle, Amani Michel Kouakou, Michael Friedmann, Vivian Polar, Clair Hershey

**Affiliations:** ^1^ CGIAR Research Program on Roots, Tubers and Bananas (RTB) led by CIP, Av. La Molina 1895, La Molina Lima Peru; ^2^ Centre de Coopération Internationale en Recherche Agronomique pour le Développement (Cirad) Avenue Agropolis 34398 Montpellier Cedex 5 Montpellier France; ^3^ International Potato Center (CIP) Plot 14, Ntinda II Road PO Box 22274 Kampala Uganda; ^4^ International Potato Center (CIP) PO Box 30709 Nairobi 00100 Kenya; ^5^ Former scientist of International Potato Center (CIP) University of Applied Sciences Bingen Berlinstrasse 109 55411 Bingen am Rhein Germany; ^6^ International Institute of Tropical Agriculture (IITA) PMB 5320, Oyo Rd Ibadan Nigeria; ^7^ International Institute of Tropical Agriculture (IITA) c/o ILRI PO Box 30709‐00100 Nairobi Kenya; ^8^ Alliance Bioversity International and International Center for Tropical Agriculture (CIAT) Rome Italy; ^9^ Alliance Bioversity International and International Center for Tropical Agriculture (CIAT) P.O. Box 24384 Plot 106, Katalima Road, Naguru Kampala Uganda; ^10^ Department of Global Development B75 Mann Library Cornell University Ithaca NY 14853 USA; ^11^ Specialized in Crop Production and Seeds Syngenta Foundation for Sustainable Agriculture 1 esplanade Jean Sauvage 49 130 Les Ponts De Ce France; ^12^ Station de Recherche sur les Cultures Vivrières (SRCV) CNRA 01 BP 633 Bouaké 01 Côte d'Ivoire; ^13^ International Consultant Flinton PA USA

**Keywords:** Banana, cassava, consumer preferences, plant breeding, potato, root and tuber crops, sweetpotato, varietal adoption, yam

## Abstract

This review of the literature on varietal change in sub‐Saharan Africa looks in detail at adoption of new varieties of bananas in Uganda, cassava in Nigeria, potato in Kenya, sweetpotato in Uganda and yams in Côte d’Ivoire. The review explored three hypotheses about drivers of varietal change. There was a strong confirmation for the hypothesis that insufficient priority given to consumer‐preferred traits by breeding programmes contributes to the limited uptake of modern varieties (MVs) and low varietal turnover. Lack of evidence meant the second hypothesis of insufficient attention to understanding and responding to gender differences in consumer preferences for quality and post‐harvest traits was unresolved. The evidence on the third hypothesis about the informal seed system contributing to slow uptake of MVs was mixed. In some cases, the informal system has contributed to rapid uptake of MVs, but often it appears to be a barrier with inconsistent varietal naming a major challenge.

## Graphical abstract

Two metrics widely used to measure the overall impact of breeding programs are: (i) adoption of modern varieties as a percentage of the total area under the crop, and (ii) the area‐weighted varietal age of a crop in a particular country or region, estimated as the average time since the release of varieties, weighted by the area across which they are adopted. The graph compares this data for roots tubers and bananas with other crops based on information gathered in 2010 (Walker & Alwang, 2015). The paper provides an update through case studies of varietal change in banana, cassava, potato, sweetpotato and yam.

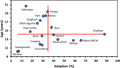

## Introduction

This article examines patterns of varietal change for roots, tubers and bananas (RTB crops), based on a literature review, and with a particular focus on sub‐Saharan Africa (SSA). The starting point is the breeders’ elicitations of varieties they consider to have been adopted and farmer surveys analysed by Walker and Alwang (2015) and followed up with case studies across the different RTB crops for those countries having the best information available on adoption. The central argument is that the importance of consumer preferences has been given insufficient attention in breeding programmes, which, until recently, have been poorly equipped to address such preferences. Indeed, consumer traits are not sufficiently considered in most varietal adoption studies. In much of SSA, farmers are among the principal consumers of the crops they grow, and, along with urban consumers, they may have quite particular needs and food types which shape those preferences. In this review, the term *consumer* is used in a broader sense to include other value chain actors, such as processors and market intermediaries, who are also concerned about post‐harvest and quality traits.

In general, varietal change is a function of multiple factors related to both the traits of varieties currently grown and those available through breeding programmes, the characteristics of growers and consumers who demand those varieties (reflected in the prices they may be prepared to pay), and the production constraints and conditions in the targeted agroecologies. The adoption of new food crop varieties in SSA has been relatively slow compared with other parts of the world. Three hypotheses are explored as shaping varietal change in RTB crops.

The first hypothesis is: *for RTB crops, insufficient priority is given to consumer‐preferred traits by breeding programmes and thus contributes to the limited uptake of modern varieties (MV) and low varietal turnover*. This is not to deny that other traits are important, too. However, the limited attention given to understanding adoption decisions around particular varieties, and the trait preferences which underpin those decisions, makes it difficult to definitively test this hypothesis. A recent review by Goddard *et al*. (2015) drew attention to the limited scientific knowledge across RTB crops around the determinants of *texture*, just one organoleptic property for consideration by breeding programmes. This article, and this special issue more generally, is a plea for more attention to understanding the nature of consumer preferences in shaping demand and driving adoption and incorporating these preferences in varietal product profiles.^1^ This is especially important in most SSA communities where farm household members are consumers as well as producers, and because of differences in roles and assets, these preferences may have a marked gender dimension.

This leads to the second hypothesis: *insufficient attention to understanding and responding to gender differences in consumer preferences for quality and post‐harvest traits has contributed to inadequately described product profiles and, hence, is also linked to slow uptake of MVs*.

In most of SSA, informal seed systems predominate in RTB crops and these may limit the opportunities for disseminating MVs. Therefore, the third hypothesis is: *the predominance of informal seed systems has been a major contributor to slow uptake of MVs*.

## Setting the scene

In the humid tropics of SSA, RTB crops ‐ principally bananas (including plantains), cassava, potatoes, sweetpotatoes and yams ‐ are the most important staple and the dominant food commodity (Lebot, 2020). Across this group of countries, the contribution of foods derived from RTB crops to total caloric needs from all sources ranges from nearly 25% in Nigeria to close to 60% in the Democratic Republic of Congo (DRC) (RTB, 2016).

Populations are still growing rapidly in SSA. By 2050 just four of the more important countries for production of RTB crops (DRC, Ghana, Nigeria, Uganda) are projected to have a combined population of 0.75 billion, reaching 1.4 billion by 2100 (UNDP, 2015). Globally, the number of people involved in RTB‐based agri‐food systems could more than double by the end of the 21st century, with most of that growth in SSA.

There are marked cultural preferences for RTB crops in SSA. The International Food Policy Research Institute (IFPRI) used the Impact Partial Equilibrium model to show that per capita consumption of RTB crops will continue to rise (RTB, 2016; Rosegrant *et al*., 2017). However, because of low productivity and market constraints, current RTB crop production in SSA does not meet basic food security needs in rural areas and is often uncompetitive with imported cereal staples from international markets for national urban consumers, resulting in missed smallholder income opportunities. As more people move to cities, value chains for RTB crops will need to improve in efficiency (e.g. higher yield per unit of input), add value and convenience, and reduce post‐harvest losses to compete with imported cereals. This will also mean changes in varietal product profiles to fit urban consumers’ demands. RTB crops will increasingly create business opportunities in both the domestic and export markets as food, feed ingredients and industrial raw materials. These crops are also likely to play a key role in food security under climate change given their flexibility and resilience (Petsakos *et al*., 2019).

## Varietal adoption and preferences of growers and consumers

### Key features of RTB breeding

Any individual plant of an RTB crop is highly heterozygous. Clonal propagation fixes this heterozygosity to allow genotypic uniformity of a variety. However, cross‐pollination by breeders (or insects) leads to wide segregation, that is, high genetic diversity among offspring. Many species of RTB crops are also polyploid with more than two sets of chromosomes, further increasing genetic complexity. While heterozygosity can be advantageous for developing a variety directly from a cross between two parents, the downside is that a particular individual cannot be replicated from any cross. Equally, it is not possible to incrementally improve an existing variety by adding individual new traits, say for disease resistance, by backcrossing. Every time a breeder makes a cross, there is wide segregation for many traits and not only for target traits. In addition, breeding for the quality traits consumers want in RTB crops is especially challenging as these traits are poorly characterised and the heritability of quality traits is either low or not yet sufficiently studied.

This complex genetic make‐up means there are almost always compromises between desired and non‐desired traits for the growers and consumers. Even when a new variety looks highly promising for many traits, a single negative trait can lead to non‐adoption. Removing that single negative trait is only possible through further lengthy breeding. It should be mentioned, however, that new tools such as gene editing are expected to expedite the management of simply inherited traits in RTB crops.

Given these complexities of breeding, once consumers identify a preferred RTB variety, that variety may be very persistent or *sticky,* in the sense that their preferences change very slowly, even in developed countries. This dynamic is well documented for potatoes where King Edward (UK), Russet Burbank (USA) and Bintje (Netherlands) continue as important varieties, each based on crosses more than 100 years old. Russet Burbank, for example, produces very oblong tubers, which store well, and are especially good for eating baked or as french fries, products for which processors have particular specifications for dry matter and sugar content. Russet Burbank still has 70% of the processed potato market in the USA (Brown, 2015).

All RTB crops are grown by vegetative propagation from a part of the plant such as its tuber, stem or vine rather than from a true botanical seed, which is used in crops such as wheat and maize. Hence, seed^2^ multiplication and distribution present special challenges due to low multiplication ratios from one generation to the next, bulkiness, perishability and pest/pathogen accumulation in planting material, all of which create further barriers for varietal replacement. In SSA, institutional constraints exacerbate these physical challenges, making the establishment of commercially sustainable formal seed systems based on routine certification difficult. As a result, almost all seed in RTB crops is recycled by the farmer from a previous harvest or acquired through informal seed systems (e.g. from neighbours or in local markets). Only a small percentage of seed is typically certified with assured precedence from released varieties (Almekinders *et al*., 2019).

Given the limited coverage of formal seed systems that provide access to released varieties, farmers may also select new varieties in three ways: (i) by accessing new genetic diversity by taking part in on‐farm trials or during demonstration trials outside of formal release processes (these varieties are known as farmer selections or *escapes*); (ii) by harvesting mutations and accidental crosses in the field, which are then multiplied through the informal seed system; and (iii) in some rare cases, by planting botanical seed and making their own selections (see e.g. de Waul *et al*., 1997; Nakagonge *et al*., 2007).

One advantage of the informal seed system in terms of potentially faster varietal turnover is that new clones do not have to compete against clean material from older releases (e.g. as in the case of very old potato varieties in the US and Europe). In developed countries, formal seed systems contribute to the slowing of varietal change as clean propagation material is widely available commercially and legislatively regulated. New clones do not compete with older clones on the quality of the propagation material, which is the same for all material regardless of vintage (Tom Walker personal communication). However, in developing countries, the amount of seed actually flowing through formal systems is usually very small, even of the modern varieties, so this advantage of clean seed is diminished.

### Key metrics of success of breeding programmes: adoption and varietal age

Two metrics widely used to measure the overall impact of breeding programmes are given as: (i) adoption of modern varieties as a percentage of the total area under the crop, and (ii) the area‐weighted varietal age of a crop in a particular country or region, estimated as the average time since the release of varieties, weighted by the area across which they are adopted. The most complete study relating to SSA using these metrics was conducted with data for 2009‐2010 (Walker & Alwang, 2015). In this study, MVs were defined either as resulting from a crossing programme or from the release of a landrace outside of its country of origin. Also included are non‐released farmer selections from breeding programmes (*escapes*). Walker and Alwang arbitrarily selected 1970 as the cut‐off date for considering a variety as *modern* and excluded releases prior to that date. This review uses the same definition of MVs and refers to *improved varieties* (IVs) as those which resulted from a purposive cross in a breeding programme. With continuing reduction in the cost of genomic analysis, more recent studies of varietal adoption have used genetic fingerprinting to definitively establish varietal provenance (Wossen *et al*., 2017). However, Walker & Alwang (2015) used breeders’ elicitations with unknown biases of actual adoption and surveys of farmers relying on their recall of varietal names but without genetic fingerprinting to validate responses. In some cases, there were significant differences between the two sources of data, which raises questions of data quality. Despite the drawbacks, their study provides a unique review of trends in breeding performance based on a multi‐country survey with a consistent methodology.

Analysis by crop type shows that RTB crops as a group have a slightly higher varietal age, reflecting a slower varietal turnover than cereals or legumes, although the difference is not enough to draw any overall conclusions (Table 1). The area under MVs is midway between the other crop groups and quite low at 32.9%. Varietal development in Africa has depended significantly on collaboration in various countries of International Agricultural Research Centres (IARC) ‐ predominantly CGIAR centres ‐ with national breeding programmes. The IARC^3^ share of varietal adoption for RTB crops in Table 1 is the highest at 74.9%, perhaps reflecting a comparative advantage for international research given the more complex genetics and less private sector interest in breeding for RTB crops compared to hybrid maize and other cereals.

**Table 1:** Adoption of modern varieties (MVs) of food crops in sub‐Saharan Africa in 2010

Crop	Total area (000 ha)	Adopted area (000 ha)	MVs (%)	Share IARC (%)	Varietal age (years)	Number of country programmes
RTB Crops/ weighted average	18 719	6156	32.9	74.9	15.2	33
Banana	916	57	6.2	34.9	10.2	1
Cassava	11 036	4376	39.7	82.5	14.1	17
Potato	616	212	34.4	90.8	19.4	5
Sweetpotato	1478	102	6.9	81.3	10.3	5
Yam	4673	1409	30.2	50.0	18.4	5
Cereal Crops/weighted average	65 936	24 257	36.8	61.9	14.4	35
Barley	971	318	32.7	23.0	18.5	2
Maize (ESA)	14 696	6470	44.0	29.4	13.0	8
Maize (WCA)	9972	6557	65.7	80.6	12.8	11
Pearl millet	14 090	2552	18.1	86.6	14.8	3
Rice	6787	2582	38.0	50.6	15.8	4
Sorghum	17 966	4927	27.4	75.0	17.4	6
Wheat	1454	850	58.5	64.5	12.8	1
Legume Crops/weighted average	23 067	7057	30.6	72.5	12.6	49
Bean	2497	724	29.0	81.0	13.8	9
Cowpea	11 472	3118	27.2	66.7	11.9	16
Faba bean	615	86	14.0	3.7	20.7	2
Groundnut	6357	1855	29.2	85.8	11.7	5
Soya bean	1185	1042	87.9	63.2	14.2	11
Other legume	941	233	24.8	85.9	16.7	6
Total/ weighted average	107 722	37 470	34.8	66.1	14.2	117

Note

IARC primarily relates to CGIAR centers but includes others. Source: Walker and Alwang (2015)

**Figure 1:**
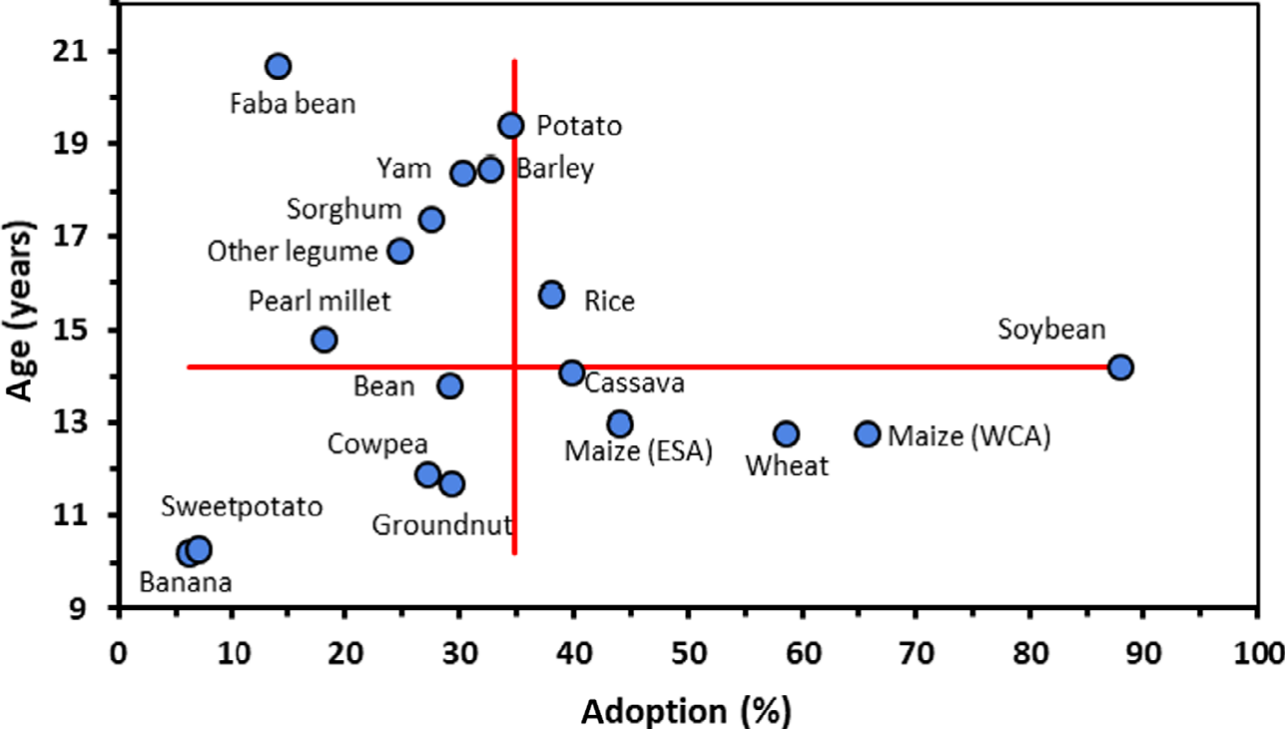
Varietal age and adoption of modern varieties in SSA in 2010. *Source*: (Walker & Alwang, 2015).

Looking at the individual crops and measuring the performance of breeding programmes by *mean varietal age* and *adoption of improved varieties* for all the countries in the 2010 study is more revealing (Fig. 1). The location of any crop in the quadrants is influenced by genetic resources available to breeding programmes, the investments into breeding and seed systems across SSA, and the challenges of breeding for any particular crop type.

The bottom right quadrant with high adoption and lower varietal age corresponds to the higher performing breeding programmes. Maize and wheat are solidly inside this quadrant, benefiting from easier genetics and prior investments in plant breeding including in developed countries in these crops. Cassava also just falls into this quadrant, supported by a strong programme at IITA whose breeders have been able to draw on prior research at the regional Amani Research station in Tanzania and the Moor plantation research station in Nigeria (Alwang, 2015). This collaboration led to the release of IITA’s Tropical *Manihot* Selection (TMS) varieties which feature mosaic disease resistance and high yields.

Potato and yam have similar adoption figures of around 30%, but in the *high varietal age* quadrant. In the case of yam, almost all MVs which comprise this figure are introduced and released landraces, such as CI8^4^ in Cote D’Ivoire. In the case of potato, adoption relates to varieties which emerged primarily from crossing programmes, gaining some additional advantage from research in developed countries and from late blight resistance work in Mexico. One contributing factor mentioned for high varietal age in potato is dis‐adoption of more modern varieties following the genocide in Rwanda, as the collapse of the formal seed system and destruction of a key research centre impeded new releases (Labarta, 2015). Walker & Alwang (2015) note a dearth of earlier research on bananas and sweetpotatoes with few releases prior to 1980. Not surprisingly, sweetpotato and banana (the latter based only on the Uganda survey) are outliers in the bottom left quadrant of Fig. 1, with low varietal age and low adoption. These results reflect young breeding programmes at the time of study and highlight the especially difficult breeding challenges for banana as a semi‐perennial crop with a sterile fruit.

The evidence from these studies presents a relatively low overall rate of adoption and a low rate of varietal turnover. Even cassava, a higher performing RTB crop, attained only 42% MV adoption. Fuglie & Marder (2015) attributes this low rate of diffusion for MVs in the study to institutional (e.g. access to seed and markets) and/or environmental (e.g. narrow adaptation to agro‐ecological conditions) barriers that may be constraining adoption.

In general, Walker & Alwang (2015) find that adoption for RTB crops is driven by varieties with higher yields and specific disease resistances – a finding that is consistent with the primary breeding targets of most RTB breeding programmes. For example, Alene (2015) notes that ‘across SSA, high yield and disease resistance are the preferred attributes of cassava varieties’; they observe the importance of good consumer acceptability, but the particular aspects or traits which underpin acceptability were not detailed. For driving higher adoption, Walker & Alwang (2015) specifically mention the importance of resistance to *Fusarium* wilt in banana; mosaic disease, bacterial blight and green mites in cassava; late blight and bacterial wilt in potato; viruses in sweetpotato; anthracnose and viruses in yam. Regarding gender differences in the adoption of varieties, there is a single reference in Walker & Alwang (2015: 178) related to the preference of semi‐subsistence producers, and especially women growers, for the Cruza 148 potato variety. This preference was due to Cruza 148’s tolerance to bacterial wilt and late blight, very short dormancy and high yields under most conditions, despite the fact that traders and consumers tended to discount this variety because of a blue ring in its flesh (Haverkort *et al*., 2015; Labarta, 2015).

Consumer preferences as a driver or inhibitor of varietal change feature less strongly and clearly in the Walker & Alwang (2015) adoption studies. The strongest recognition for the importance of consumer preferences in constraining adoption in their studies relates to low adoption rates of cooking, dessert and beer bananas in Uganda. Thus, they conclude: ‘Stimulating varietal change in a clonally propagated crop – and one that is not an annual – is a challenging proposition anywhere in the world. A focus on disease resistance is necessary, but entrenched consumption preferences are potentially major constraints to adoption’ (Walker *et al*., 2015).

Other studies mention the importance of consumer preferences in driving adoption of particular varieties. An outstanding example is the C18 yam variety, ‘known for making tasty yellow porridge.’ After its introduction in Côte d’Ivoire in 1986 (CNRA, 2016), C18 yams expanded rapidly, becoming, in some locations, the most cultivated variety of *Dioscorea alata* – or ‘water’ yam (Walker *et al*., 2015). The Kinigi variety of potato (selected by ISAR from a CIP cross) provides another interesting example, as it gained ‘wide market acceptance for processing characteristics and as a table potato’ and continues as the preferred variety in Rwanda even though its late blight resistance has broken down. In the case of sweetpotato, the old *Tanzania* variety has been widely adopted in East African countries. Originally found in the sweetpotato local collection at Serere station in Uganda, and subsequently selected at the agricultural research station at Amani in Tanzania in the 1950s, the *Tanzania* has drought tolerance and virus resistance in a yellow‐fleshed ‘mealy background preferred by consumers’ (Labarta, 2015). For cassava, consumer preferences in the DR Congo drove adoption of the *Sadisa* (TMS91/203) variety, which covered 14% of the cassava area. In addition to its high yields and resistance to green mite and bacterial blight, *Sadisa* has ‘high dry matter and high‐quality flour (cream)’ (Alene *et al*., 2015).

More recent studies which use genotyping‐by‐sequencing (GBS) markers to precisely identify varieties by the genetic fingerprints for RTB crops are, to date, only available in SSA for cassava in Nigeria (Wossen *et al*., 2017) and Ghana (Rabbi *et al*., 2015), and for sweetpotato in Ethiopia (Kosmowski *et al*., 2018). The next section of the paper looks at contrasting case studies of varietal change in five different RTB crops to understand the underlying drivers and, in particular, the importance of consumer preferences. One caveat is that in few cases nationally representative farm level studies were available. Consequently, to provide a richer picture of the dynamics of adoption, information was used from local level adoption studies and personal communications.

## Case studies of varietal change

### Hybrid banana varieties in East and Southern Africa

Bananas (including plantains) are the most complex RTB crop to breed and also have very marked consumer preferences. Bananas in Eastern and Southern Africa are consumed in three primary modes (with many variations): green (unripened) for cooking, the most important use; banana beer or juice; and as dessert banana. Between 1986 and 2016 more than 44 hybrid (i.e. from a deliberate cross or improved varieties) banana varieties were introduced in Kenya, Mozambique, Rwanda, Tanzania and Uganda for evaluation and dissemination Karamura *et al*. (2016). Of these varieties, 18 were not adopted. While farmers valued the resistance to pests and diseases and early maturation for all three product types, the sensory attributes and other consumer preferences also featured highly in adoption criteria. For *green‐cooking bananas*, consumers preferred sensory attributes, such as taste, flavour, texture and colour after cooking. For *beer/juice‐*making, different varieties were preferred depending on consumer preferences for astringency, starch and bunch size. And for *dessert bananas* in local and regional markets, consumers preferred sensory attributes while farmers selected new varieties having bunch, hand and finger characteristics similar to traditional dessert varieties (Karamura *et al*., 2016).

A survey of 800 households from Uganda and the Kagera region of Tanzania showed that 9% of households in Uganda and 19% in Tanzania had adopted improved (hybrid) cultivars of FHIA01, FHIA03, FHIA17, FHIA23 and an elite endemic (*Mpologoma*). The average share of the banana mats allocated to improved cultivars was 13% in Uganda and 34% in Tanzania (Smale & Tushemereirwe (2007). Adoption of hybrids in the Kagera region of Tanzania was greater than Uganda due to stronger dissemination and the perception among farmers that hybrids were superior to landraces in terms of yield and resistance to pests (e.g. weevils) and disease biotic pressures (e.g. black sigatoka). In both locations, farmers ranked hybrids higher than landraces for all traits, except one – quality for cooking (especially in Uganda). The study noted that within the household the gender of the banana production decision‐maker reflected preferences for banana attributes. Women tended to prefer cultivars destined for consumption while men were more likely to use hybrids, because of their suitability for brewing beer. Hence, Smale & Tushemereirwe (2007) noted that because cooking is the most important driver of adoption of FHIA hybrids, increased adoption rates further appeared unlikely to happen without changes in consumer preferences.

In Uganda, at least 31 hybrid varieties were introduced between 1990 and 2011. Six had been officially released and 25 only disseminated informally. A survey of 240 households in four regions showed 57% of farmers had at least one mat of hybrid varieties but the adoption intensity (considering the number of mats with hybrid varieties) was only 6% (Kagezi *et al*., 2012). The two most adopted varieties were KABANA 3 (FHIA 17) and KABANA 6H (M9 – or NARITA 7), the latter of which offered resistance to weevils, nematodes and Black Sigatoka (Kagezi *et al*., 2012). Fewer farmers had at least one mat of NARITA 7 but it was equal to FHIA 17 in adoption intensity at 1.6%. By 2016, NARITA 7 was more rapidly adopted than other MVs (Wamboga‐Mugirya, 2016). Akankwasa *et al*. (2013) found that M9 was the most preferred hybrid variety. However, many of the respondents preferred the local variety *Mbwazirume* for its good taste, large bunch size, soft texture and good flavour – the most desirable attributes. While M9 could not compete on taste and flavour, it was preferred for food security in some contexts because of high productivity. More recently, new hybrids with similar agronomic properties and productivity and improved culinary traits nearly indistinguishable from preferred Ugandan landraces, have been released (Tumuhimbise *et al*., 2018).

The overall conclusion from this case study is that it is challenging to introduce a banana hybrid unless it can meet the marked consumer preferences for one of the product types. Otherwise, adoption will be quite limited despite the banana hybrid having high yield and other favourable agronomic traits.

### Cassava in Nigeria

Most cassava in Nigeria is produced and processed locally into *gari* (grated, fermented and toasted to a dry granular flour), *fufu/akpu* (fermented wet paste then cooked with additional water) or *lafun* (dried chips ground into flour and cooked into paste). A small percentage is sold to larger factories for flour or starch production. An even smaller amount is consumed fresh at home as boiled or as boiled‐then‐pounded. Almost all processing is done by women at home or in specialised processing centres in rural areas (Curran *et al*., 2009; Teeken *et al*., 2018). Many cassava varieties in Nigeria have a relatively high cyanogen content (referred to as *bitter*) and can be toxic if consumed after boiling, but the cyanogen levels are reduced to safe levels by processing. Cassava varieties consumed fresh usually have very low cyanogen content and are called *sweet* or *poundable* varieties (Nweke, 2004).

The International Institute of Tropical Agriculture (IITA), in collaboration with national partners such as National Root Crops Research Institute (NRCRI), began cassava breeding in the early 1970s focusing on resistance to cassava mosaic disease (CMD) and cassava bacterial blight (CBB). Other breeding traits included high yield, good root quality, high dry matter, low cyanogen content and resistance to lodging (Wossen *et al*., 2019). This work led to a sustained deployment of CMD and CBB‐resistant cassava varieties (Dixon *et al*., 2010; Alene & Mwalughali, 2012). Farmer trials with a diverse set of clones were conducted widely across Nigeria (see for example Akoroda *et al*., 2004). A varietal adoption survey in Nigeria in 2009 based on expert elicitation reported 46% MV adoption, nearly all of which were either IITA crosses or from partners that used IITA parents (Alene *et al*., 2015). The variety TMS 30572 was reported as the most widely adopted MV by far with an adoption area of 17.8%. This variety combines high yield and medium CMD resistance but also is good for making *gari*. Komolafe & Arawande (2010) confirmed this finding noting that TMS 30572 had the highest carbohydrate content and *gari* yield on fresh root basis compared to two popular landraces. *Gari* from TMS 30572 also had the highest swelling index in cold water and the highest bulk density. Organoleptic assessment showed high acceptability for TMS 30572 as *gari*, similar to the landraces.

The 2015 survey based on genetic fingerprinting showed a 39.9% adoption rate of MVs using the same definition of MV as the 2009 study (Table 2). This rate is slightly lower than the figure estimated in 2009, with a mean varietal age of 17.4 years based on expert opinion (Tesfamicheal Wossen personal communication).^5^ The 2015 survey showed an adoption rate of 7.7% for TMS 30572, making it still the most important single MV grown in Nigeria. However, this was substantially below the adoption figure reported in 2009, likely reflecting an earlier overestimate, and, perhaps to a lesser extent, gradual replacement by more recent releases. The discrepancies for adoption based on genetic fingerprinting compared to the earlier study for several varieties reflects the challenges of expert identification. However, the data also showed a more strategic oversight. Farmer selections (escapes) that had not been released as varieties made up more than half of the area under MVs and were comprised of two types: (i) non‐released improved clones that had been propagated through the informal seed system, and (ii) clones that were genetically similar to improved materials, either as clones or released varieties. Type 2 materials were probably derived from accidental cross‐ or self‐pollination from improved materials, resulting in seeds that germinated in farmers’ fields and which they selected and propagated (Wossen *et al*., 2017). Bredeson *et al*. (2016) note that farmer varieties in SSA include specific *M. glaziovii* haplotypes (a source of CMD resistance) introduced from organised breeding programmes, reflecting the importance of farmer selections. The prominence of unreleased material, and especially those of type 1, indicates that breeding trials and other breeding activities are important sources of variety dissemination by farmers and they do actively propagate suitable material.

**Table 2:** Economically important improved cassava varieties in Nigeria

Type	Variety	Year release	2009 adoption (% area)	2015 adoption (% area)
IV	TMS 30572 (Nicass 1)	1984	17.8	7.7
IV	TMS 4(2)1425 (Nicass 2)	1986	8.7	0.1
IV	NR 8082 (Nicass 14)	1986	7.2	0.4
IV	TMS 92/0326 (Nicass 27)	2006	2.8	0.0
IV	TMS 30555 (Nicass 10)	1976	2.4	0.1
IV	TMS 98/0581 (Nicass 24)	2005	2.0	0.6
IV	TMS 98/0505 (Nicass 22)	2005	1.8	0.3
IV	TMS 50395	1986	0.0	1.8
IV	TMS 011368	2011	0.0	0.7
IV	Other released IVs		0.0	3.3
IV farmer selection	Improved clones not released	n.a.	n.a.	16.8
IV derived	Genetically similar to IVs	n.a.	n.a.	6.7
Landrace released	TME 419 (Nicass 20 ‐release)	2005	2.8	1.5
	All MVs (National)		46.0	39.9

Note

Estimate for 2015 is corrected to allow for intercropping. It includes the landrace as TME 419 which originated from outside Nigeria, but excludes released Nigerian landraces such as MS‐3 (Odongbo) and MS‐6 (Antiota) which have adoption areas of 7.2%, including these would increase adoption area in 2015 to 60.3%. Other released IVs refers to officially released MVs from crossing programs such as TMS 92/0057, TMS 97/2205, TMS 97/2205, TMS 98/0002 not listed in the table. Source: Alene *et al*. (2015) and Wossen *et al*. (2017)

The 2015 adoption study of 2500 households in Nigeria found that over 70% of farmers considered consumer traits such as white root colour and quality of *gari* or *fufu/akpu* as very important, as well as root yield, big root size, early maturity, ability to store in ground, and pest and disease resistance (Wossen *et al*., 2017). For *gari* alone, taste was the most important consumer trait for most farmers, while for *fufu* taste was second. The study showed that the quality of *gari* and *fufu*, as well as early maturity, root yield and storability, have statistically significant effects on the adoption decisions for spouses and heads of household. For spouses (mostly women) suitability for pounding affected their decisions, partly reflecting a preference for non‐bitter cassava (Wossen *et al*., 2017) but also possibly highlighting the importance of the less visible occasional fresh consumption (which demands no processing) for household food security.

Wossen *et al*. (2017) concluded that breeding programmes need to target consumer traits such as quality of *gari* and *fufu*, market preferences such as white flesh colour, and production traits of early maturity, pest and disease resistance, and high root yield. However, Bentley *et al*. (2016) was less clear on the importance of particular quality traits, with some focus groups noting that all or most cassava can be made into *gari*, depending on how you toast it. So, it may be that skilled processors can compensate in part for the difference in quality in the cassava roots they source.

The most important gender difference on trait preferences reported by Wossen *et al*. (2017) who found that women want cassava that is easy to peel. This finding was not reported in the study by (Teeken *et al*., 2018).

Many farmers grow several varieties including landraces. This choice reflects farmers' desire to maintain a set of different cassava varieties with contrasting agronomic and consumer traits to meet different needs. There is clear evidence of farmers retaining landraces to meet specific consumer preferences. Teeken quoted women in a focus group study, who said ‘*Nwaocha* is good for food products such as *abacha*, *akpu* and *gari* due to its fine white colour. Unlike *gari* and *akpu* that may sometimes have dull white colour, *abacha* [grated manually into long thin parts, partly cooked and then fermented] must have bright white colour which can only be got from *Nwaocha* variety.’ (Teeken *et al*., 2018). *Nwaocha* was especially preferred by women because it ferments quickly in 2–3 days, is odourless, and has good product quality for *abacha, lafun* and *gari*. These focus group studies also found that women more often expressed product quality for *gari*, *eba*, *fufu* and *lafun* as a criterion in ranking varieties while men more often expressed agronomic traits. Teeken *et al*. (2018) note that cooking/processing traits are important especially for women and should be given more importance in breeding programmes.

Farmers may prefer bitter types of cassava because they are less prone to being dug up by rodents, monkeys or wild pigs (Pircher *et al*., 2019)^6^ as reported in earlier studies (Cock, 1982). In Malawi, farmers expressed marked preferences for bitter types (Chiwona‐Karltun *et al*., 1998). So those same farmers may also prefer sweet (non‐bitter) varieties for some uses such as for quick boiling to feed workers in south‐west Nigeria (Teeken *et al*., 2018) or for planting close to the homestead where goats can be poisoned by eating the bitter types and wild animals are not a threat. Nweke (2004) notes that most cassava grown in the early 20th century were the sweet type that could be eaten fresh but gave low yield and were susceptible to pests and diseases. As populations increased and cassava was transformed during the 20th century from a staple to a cash crop, farmers planted more bitter types which tended to have higher yields (Nweke *et al*., 1994). Dufour proposes that the spread of CMD from the 1930s may have hastened the switch to bitter types, which had more CMD resistance reducing availability of specific types suitable for boiling (Dominique Dufour personal communication).

This case study of cassava confirms the importance of consumer‐preferred traits in influencing adoption decisions, with suitability for *gari* considered a key trait in the widely adopted TMS 30572 variety (although other traits, including disease resistance, are important, too). The complexity of processing options for cassava in Nigeria, high heterogeneity for trait preferences, and the difficulties around varietal identification make cassava one of the most challenging cases to unpack and detail the importance of consumer traits. Nonetheless, given the scale of cassava production in Nigeria, analysing and understanding these preferences is extremely important.

### Potato in Kenya

Until the 1970s most MVs of potato grown in Kenya were of European origin. For example, with support from a plant breeding institute in Scotland, *Kenya* and *Kenya Baraka* were released in 1973, ostensibly with horizontal resistance to late blight (Robinson, 2007). When CIP’s breeding programme in East Africa began in the 1970s it also focused on introducing varieties with late blight resistance, which was perceived as the highest priority trait. Dr John Niederhauser’s search for late blight resistance in Mexico began in the 1940s, and by the late 1960s three late blight resistant varieties had been approved for release in Mexico. Distributed to East Africa, these varieties were the starting point for CIP’s potato improvement programmes (Labarta, 2015). More recently, the *Asante* and *Tigoni* varieties released in 1998, and *Sherekea* released in 2010, were selections made by the Kenyan national programme from CIP crosses that offer moderate resistance to late blight and include many important consumer traits.

Potato in Kenya is an important cash crop. Farmers have close connections with traders. In Meru county, potato has long been a major cash crop and a series of farm level surveys make it possible to construct a long‐term picture of varietal change based on farmer identification (Table 3). After a long period of little varietal turnover, a more dynamic picture of varietal change in recent years emerges (the shading in the table shows the period over which varieties were encountered). The *Kerr’s Pink* variety was introduced in the 1920s and for around 50 years, it was the only potato grown in Meru (Durr & Lorenzl, 1980). *Kerr’s Pink* was so common some even called ‘*Meru*’ potato. Farmers explained to McArthur Crissman (1989) that *Kerr’s Pink* was the only potato they could sell to traders unless supplies ran short. A few farmers grew varieties from farmer selections called *Nyayo* and *Ngure*. But most still stuck to *Kerr’s Pink* despite high susceptibility to late blight because it attracted a premium price. By 2005 *Ngure*, a higher yielding and early maturing farmer selection, became the most widely grown variety in Meru. *Ngure* was highly preferred by traders and fetched a higher price than *Kerr’s Pink* although the two red‐skinned tubers look similar. A short time later, *Asante*, another red‐skinned variety that originated in CIP, gained sudden popularity. Based on these varieties, it can be concluded that red‐skin colour is a major driver of adoption in Meru, which may likely be derived by its visual association to *Kerr’s Pink*. However, in other parts of Kenya, white skin varieties have prevailed since the 1980s.

**Table 3:** Potato varieties in farmer’s fields in Meru between 1976 and 2017 (percentage of sample farmers)

Variety	Skin colour	Maturity^f^	Late blight 1‐9^g^	Year Release Kenya	Provenance	1976^a^	1988^b^	2001^c^	2005^d^	2017^e^
Kerr’s Pink	Pink	Med	V. sus.	1927	Engl/KALRO	100	93	50	20	0
Dutch Robijn	Red	Med	7.54	1945	Holl/KALRO	0	0	0	0	2
Desiree	Red	Early		1972	Holl/KALRO	0	11	0	12	0
Kenya Baraka	White	Late		1973	Scot/KALRO	2	4			
Roslin Tana	White	Med		1974	Scot/KALRO	2	7	0	0	0
Ngure	Red	Med	Sus.	n.a.	Farmer	0	36	84	67	0
Nyayo	White	V. early		n.a.	Farmer	0	6	0	6	0
Asante	Red	Early	6.46	1998	CIP/KALRO	0	0	20	22	40
Tigoni	White	Med	6.26	1998	CIP/KALRO	0	0	1	5	0
Komesha	Red	Med. late		n.a.	CIP/KALRO	0	0	0	8	0
Tigoni red	Red	Med	8.99	n.a.	Farmer	0	0	0	30	0
Sherekea	Red	Late		2010	CIP/KALRO	0	0	0	0	20
Shangi	White	V. early	7.05	2015	Farmer/KALRO	0	0	0	0	32

Early ≤3 months, medium 3–4 months, n.a., not available.

*Source*: ^a^Durr & Lorenzl (1980), ^b^Crissman *et al*. (1993), ^c^Nyankanga *et al*. (2004), ^d^Kaguongo *et al*. (2008) and ^e^Sinelle (2018), ^f^Elmar Schulte‐Geldermann, personal communication, ^g^Forbes (2012); Hortinews (2018).

*Shangi* is a farmer selection that is very likely a clone developed by CIP for on‐farm testing; since 2005 its adoption in Kenya has expanded rapidly.^7^ *Shangi* is a quick maturing, white‐skinned potato with almost no dormancy and highly preferred by traders. *Shangi* entered Meru sometime in 2010. By 2017, 32% of farmers sampled were growing *Shangi*, making it the first white‐skinned variety with significant adoption in Meru (Sinelle, 2018). Nevertheless, red‐skinned varieties are still highly preferred, and small‐scale seed producers in Meru say farmers still want *Asante* and *Sherekea* (Monica Parker personal communication). However, in the rest of Kenya, *Shangi* was adopted massively, and by 2017, covered an estimated 60% of the cropped area surveyed by Sinelle (2018). *Shangi’s* good cooking quality (fresh and for chips), quick cooking time (when boiled) and its large tuber size, high yields and early maturity drove high adoption rates among consumers and farmers. The tuber size, yield and low dormancy, and early maturity make *Shangi* suited to year‐round production where it can be immediately planted (Sinelle, 2018).

Varietal change in Meru from 2017 (Table 3) also shows how seed supply (or lack of it) is a factor in adoption. Kisima Farms, the major commercial producer of certified seed, consciously withdrew *Asante* and scaled up production of *Sherekea* due to its comparative ease to produce and profitability, which mirrors the growing share of that variety in Meru (Ian Barker personal communication). *Sherekea* is also superior with late blight resistance and a better fit with consumer preferences.

In 2017, no differences were found between men and women surveyed in terms of reasons for adoption or dis‐adoption of varieties in Meru or the other locations surveyed (Sinelle, 2018).

Overall, this case study from Kenya shows a dynamic pattern of recent varietal change with increased area under MVs and declining varietal age. The informal system played a significant role in Kenya with one widely adopted farmer selection. Commercial seed producers also enabled adoption of released varieties and consumer preferences featured strongly as an underlying driver.

### Sweetpotato in Uganda

Sweetpotato is grown by most Ugandan rural households, predominantly by women. Storage roots are usually consumed fresh – boiled or steamed – for daily family consumption with limited sales of the fresh roots in urban markets (Bashaasha *et al*., 1995; Gibson *et al*., 2008). Generally, adult consumers prefer white or yellow‐fleshed sweetpotato with a high dry matter content and ‘mealy’ texture.

Sweetpotato research was conducted at Serere in Uganda and at the Amani Centre in Tanzania during the 1960s. Their work led to the selection of the ‘*Tanzania*’ variety, a yellow‐fleshed landrace that was widely adopted in East Africa, and preferred by farmers and in markets (Labarta, 2015). During the 1990s, *Tanzania* became a dominant variety for growers in Soroti district who sold their produce in Kampala. In fact, *Tanzania* was called ‘Soroti potato’ by Kampala traders. By 2013 in Soroti, *Tanzania* had succumbed to virus pressure and was displaced by *Boy*, another yellow‐fleshed variety, possibly a farmer selection, with a similar storage root but inferior in terms of cooking quality. Traders began referring to *Boy* as ‘Soroti potato’. Farmers were interested in purchasing certified vines of *Tanzania,* because they felt roots would be appreciated by consumers in Kampala for its superior culinary quality but only orange‐fleshed sweetpotato (OFSP) varieties were available as certified (Graham Thiele personal observation).

In Uganda and all of SSA, the CIP sweetpotato breeding programme in SSA began in 2000. Prior to that time, CIP was distributing crosses made elsewhere (Labarta, 2015). In collaboration with national programmes, CIP placed major focus on introducing beta‐carotene as a key post‐harvest trait in a background with good virus resistance and high dry matter content (although this is not a standalone consumer‐preferred trait). These OFSP varieties, when disseminated with appropriate nutrition education, can significantly contribute to reducing vitamin A deficiency. Later, CIP began working to modernise sweetpotato breeding, strengthening national breeding programmes in East Africa and broadening access to adapted germplasm. This work led to accelerated releases across the region, and to a large expansion in access to MVs in Uganda.

The Ugandan national sweetpotato programme at National Crops Resources Research Institute (NaCCRI) has long been one of the strongest in SSA. In 1995, *Sowola* was released, featuring improved virus resistance; it had been selected from crosses that included preferred local landraces as parents (Mwanga *et al*., 2001). By 1999, NaCCRI was making crosses to feature beta‐carotene as a target trait and released NASPOT 5. In 2004, NaCCRI released two OFSP cultivars – *Ejumula* and SPK004 (*Kakamega*) – selected from landraces (Mwanga *et al*., 2007). OFSP varieties NASPOT 9 O (*Vita*) and NASPOT 10 O (*Kabode*) followed in 2007, and 6 years later, NASPOT 12 O (*Gerald*) and NASPOT 13 O (*Joweria*) were developed from crosses using CIP parents (Labarta, 2015; Mwanga *et al*., 2016). NASPOT 8 was also released in 2007 and became the most popular NaCCRI OFSP variety. It has preferred eating characteristics, confirming the importance of consumer traits in adoption, but it did not meet the criteria for minimum B‐carotene levels across environments, and thus was not promoted under the HarvestPlus program (Mwanga *et al*., 2009).

By 2010, released landraces in Uganda accounted for approximately 9.0% of the sweetpotato area in the nationally representative farmer survey – much lower than expert estimates (breeders’ elicitations). White and yellow varieties from the national cross programme covered 6.4% of the area, and nearly all of that was NASPOT 1, known for its high dry matter content (Labarta, 2015). Labarta *et al*. (2012) suggest that part of the discrepancy with expert estimates is because dis‐adoption of improved varieties may have occurred in the north of Uganda where vine distributions were not accompanied with support on how to conserve the vines during prolonged dry periods. The authors observe that ‘having over 300 of local landraces in Uganda that have adapted to the very diverse local production conditions make it difficult for many improved varieties to replace them’. Rachkara *et al*. (2017) note that free government and NGO distribution of vines in Uganda may have inadvertently undermined the capacity of a functioning informal seed system, reflecting a deeper structural issue regarding the way new varieties are distributed, which works *against* rather than *with* the informal system.

Large dissemination campaigns in Uganda that provide access to vines and target rural consumers with health messages about the value of OFSP initially led to a geographically‐focused adoption of OFSP (Arimond *et al*., 2010). OFSP varieties NASPOT 9 O, and NASPOT 10 O together had 1.5% area coverage, and the OFSP landrace *Ejumula* covered 1.1% of area nationally. However, 8% of farm households cultivated some OFSP varieties, usually alongside white or yellow. These findings of relatively small areas planted to OFSP by adopting households are consistent with the bio‐fortification message that the consumption of small quantities of beta‐carotene‐rich varieties can overcome vitamin A deficiency, particularly among the most vulnerable primary target groups: pregnant and lactating mothers and children under five.

During a participatory plant breeding that led to the release of NASPOT 11, farmers were asked to list their preferred attributes for OFSP. Four of the top ten attributes were consumer traits: mealy, non‐fibrous, attractive colour of roots, and ‘sweet’ when cooked. Other key attributes included good root yield, big roots, early root maturity and continuous root yield for piecemeal harvest (Gibson *et al*., 2011).

While documentation on varietal releases suggests the importance of consumer traits the national farmer survey of varietal adoption gave the topic scarce consideration, so overall only limited support is provided for Hypothesis 1 (Labarta, 2015). With regard to Hypothesis 2, low levels of adoption and dis‐adoption may have been partly related to a failure to engage functioning local informal seed systems.

### Yam in Côte D’Ivoire

Lack of knowledge of genetic diversity, scarcity of flowering, difficulties of crossing and inadequate investment have limited progress in breeding yams (Arnaud *et al*., 2017). Most varietal adoption in West Africa has been through the introduction and distribution of landraces of clones of water yams (*D. alata)* from Asia and the South Pacific as an alternative to white guinea yams (*D. rotundata)*. In Côte d’Ivoire in 1971, CIRAD (formerly IRAT) introduced the *Florido* variety, a *D. alata* landrace, from the Mayaguez germplasm in Puerto Rico, to Côte d'Ivoire (Martin & Rhodes, 1973). *Florido* comes from a group of cultivars highly appreciated in Vanuatu and known for short cylindrical, rounded tubers with white flesh, and crumbly when cooked (Arnaud *et al*., 2017). This variety was introduced as part of a plan to mechanise harvests due to smaller tuber size and shape, (Rodriguez, 1983; Doumbia *et al*., 2004). The mechanisation plan failed, but from 1978 *Florido* was widely adopted because of its high yields, good adaptation to low fertility soils, resistance to internal brown spot disease and wilting, and ease of propagation.^8^ It largely displaced the *Bétè‐bètè* variety, which was a much earlier *D. alata* introduction (Doumbia *et al*., 2004). Farmers adopted *Florido* mainly as a cash crop and used it to replace longer standing water yam landraces introduced centuries ago. Consumer preferences played a large role in adoption as people in major cities preferred eating it fried as fast food (especially among low income consumers). Unlike most water yams, *Florido* can be used for preparing *foutou*, a common local dish (Doumbia *et al*., 2004). Farmers continued to cultivate white guinea yams and, in particular, the *Krenglè* and *Kpouna* varieties, which were preferred for excellent organoleptic quality when pounded. Moreover, white guinea yams are more difficult to grow and need more fertile soils than water yams, and are generally cropped after long‐duration fallow after slash and burn of forest. By 1996, *Florido* was reported as occupying about 41% of the total area under yam (Table 4).

**Table 4:** Adoption surveys of yam showing modern varieties in Côte D’Ivoire

Year	Variety and area		Coverage region	Type	Source
1996	Florido	41%	Dabakala	Farmer survey	Doumbia *et al*. (2004)
	Florido	22%	Dikodougou		
2007	C18	49%	Belier (Toumodi)	Farmer survey	Doumbia *et al*. (2014)
	Florido	34%			
2009	C18	61%	National	Expert estimate	Alene *et al*. (2015)
	C20	9%			
	TDR205	2%			
	TDR608	2%			
	NDRBD10	1%			
2017	Florido	65%	Gontougo	Farmer survey	Kouakou *et al*. (2019)
	Florido	40%	Boukani		
	C18	15%			
	Florido	18%	Gbèkè		
	C18	17%			
	Florido	21%	Goh (Gagnoa)		
	C18	16%			
	Florido	16%	Poro		

In 1986, another selected *D. alata* landrace – C18 – was introduced from Cameroon. C18 seems to have been a farmer selection from the experimental station and spread from farmer to farmer before its official release in 1998 (Doumbia *et al*., 2014; Alene *et al*., 2015). During group interviews, farmers noted that C18 was high yielding, stored well, showed complete resistance to brown spot and made better *foutou* than Florido and other *D*. a*lata* varieties. Women noted that C18 was easy to prepare, either boiled or stewed, and made good *foutou* (Doumbia *et al*., 2014). However, field trials for C18 showed that it yielded less than other *alata* clones, which had not been widely grown. This difference confirmed the importance of culinary quality in adoption (Kouakou *et al*., 2012). As (Doumbia *et al*., 2014) notes, by 2007 in the central region of Côte d'Ivoire" the average coverage per farm was as follows: C18 covered 0.17 ha, Florido 0.12 ha, all *D*. A*lata* (0.32 ha), and all yams (*D*. *alata* and *rotundata*) 0.35 ha. Alene *et al*. (2015), estimated (based on expert opinion) that by 2009, C18 had 61% of the total area cropped to yam. However, this estimate is almost certainly inaccurate, because the survey entirely neglected *Florido*, which continues to be an important yam together with C18 (Dufour personal communication). A recent study by Kouakou *et al*. (2019), which covered a larger and more diverse area than Doumbia *et al*. (2014) showed that C18 still appears to trail behind *Florido* in adoption in the regions surveyed.

Good culinary quality was key to the successful and relatively rapid adoption of *Florido* and then C18. Nevertheless, Ivorians mainly prefer *D. rotundata* varieties as they produce a good pounded yam. They preferred *D. rotundata* over *D. alata* varieties in sensory tests which included Florido (Nindjin *et al*., 2007). But even if *D. rotundata* varieties are preferred, they are much more expensive in urban markets (up to 400% higher) than *D. alata*. Also, after storage, *D. alata* is a better pounded yam and so can be used during the periods of shortage.

## Major findings

This review had two principal limitations. The first was the lack of national or large‐scale farmer surveys on the adoption of varieties in RTB crops. In general, these types of surveys have been rare in SSA, but this review drew on those that were available. The second limitation is that this review was based on case studies of particular crops each in one country where more adoption information was available. A complete study across SSA would not be possible within the remit of a single article given the complexity of the change processes. Nevertheless, in general, many of the findings in single countries resonate with dynamics in other countries in the region where similar drivers and trends operate. So, with these caveats, the following conclusions may be drawn.

In general, MVs for RTB crops in SSA have a relatively low rate of adoption that has likely increased in recent decades. Each of the RTB crops reviewed in this study shows a different set of trends for varietal turnover:

- Banana has been the most challenging of all crops with a very low adoption rate, especially for hybrids (i.e. from deliberate crosses). While these improved varieties have incorporated resistance to leaf spot and are higher yielding, they usually do not offer the particular organoleptic characteristics consumers are looking for across different banana products.
- Sweetpotato has also shown a relatively low rate of varietal adoption, although stronger breeding programmes from the 1980s have contributed to a growing number of releases and a consequent rise in MV adoption. This rise may be expected to continue after renewed investment in sweetpotato MVs starting in 2000 and large dissemination efforts since 2010. CIP has emphasised high beta‐carotene as a key trait, with a background of virus resistance, drought tolerance and the mealiness consumers prefer. However, high beta‐carotene in the absence of nutrition education is not a consumer‐preferred trait and may limit adoption.
- Yams have shown a higher rate of adoption with a median varietal age, but this has been linked primarily to the introduction of *D. alata* varieties and less to bred varieties (although there are promising IVs in the pipeline).
- Potato MVs have had higher adoption but a relatively high varietal age. Progress with potatoes has been limited by dis‐adoption of more recent varieties in Rwanda, which offset higher rates of adoption in Kenya. It is probable that the extensive adoption of the *Shangi* variety in Kenya, a relatively recent release and most likely a farmer selection from clones originating from CIP, and enhanced adoption of modern market‐preferred varieties like *Sherekea,* promoted through commercial seed systems, have lowered mean varietal ages and increased MV adoption.
- At the time of the 2009 survey, cassava was the most successful breeding programme in terms of adoption of MVs and lower varietal age, in part because the IITA breeding programme was able to build upon prior breeding programmes in SSA. More recently, genetic fingerprinting in Nigeria confirmed that approximately 40% of coverage under MV, but this high figure was owed more to farmer selection than varietal release.

The case studies provided the following answers to the three hypotheses considered in this review:

Hypothesis 1 For RTB crops the insufficient priority given to consumer‐preferred traits by breeding programmes contributes to the limited uptake of modern varieties (MV) and low varietal turnover.

In all case studies, there was at least some evidence of consumer preferences as drivers of adoption, although other traits are important too. In the cases of yam, cassava and potato, favourable consumer characteristics enabled adoption, and in the case of banana, unfavourable consumer preferences for hybrids most likely limited uptake. For yam (e.g. C18’s suitability for *foutou*) and potato (e.g. *Shangi’s* short cooking time), key consumer preferences helped drive rapid varietal turnover when linked to favourable agronomic traits. This evidence provides a strong confirmation for the hypothesis that one of the major factors slowing varietal turnover has been insufficient attention in breeding programmes to consumer preferences.

Lower rates of varietal turnover also mean that landraces predominate – as is clearly the case of yams, bananas and sweetpotato. Cassava was more evenly balanced. For yams, two of the more successful MVs ‐‐ *Florido* and C18 – are both introduced *alata* landraces. For cassava, some recent successful MVs have been selected from landraces (e.g. TME 419 in Nigeria). Replacing a successful RTB landrace that has marked consumer preferences is not easy. This situation is likely changing already with improved genomic information and gene manipulation technologies, but there will be a lag between release and adoption of improved varieties in the breeding pipeline. Breeding programmes may improve performance and varietal turnover if they are able to address the key consumer traits using the five‐step methodology for developing food product profiles, as proposed by the RTBFoods project (Forsythe *et al*., 2020).

Farmers are also growing a portfolio of varieties for different production uses and/or environments. Sometimes trait preferences for different varieties in the portfolio may be completely different (e.g. farmers may want both bitter and sweet cassava), so the question of which they prefer is not straight‐forward, even though surveys and focus group discussions frequently ask it.

Specific seed traits are also important in RTB crops. This was most apparent for potato in Kenya, where *Shangi* has a clear advantage in terms of year‐round availability because of very short dormancy. In cassava, the straight stems of TME 419 provide one advantage as they are much easier to bundle, store and transport. For yam, Florido is preferred in part because a smaller piece of the tuber can be used for propagation compared to other varieties.

Hypothesis 2 Insufficient attention to understanding and responding to gender differences in consumer preferences for quality and post‐harvest traits has contributed to inadequately described product profiles and, hence, is also linked to slow uptake of MVs.

It is not possible to address this hypothesis fully from the evidence of the case studies in this paper. Gender differences were not considered in the case of yam. In the case of banana it was explicitly noted that gender influences trait preferences (Smale & Tushemereirwe, 2007). For potato, Labarta (2015) mentioned women’s preferences for the *Cruza* variety as a more robust type suitable for home consumption. However, in Sinelle’s Kenya study (2018), no differences were found between women’s and men’s reasons for adoption or dis‐adoption of varieties, but specific trait preferences were not considered. Where attention was given to gender in the case of cassava, differences were found regarding cyanogen content, ease of peeling and preferences for particular varieties such as *Nwaocha* for making *abacha*. Overall, findings from the case studies provide some support for the hypothesis that insufficient consideration of gender differences has contributed to inadequately described varietal product profiles. Women are often involved in the preparation and processing of RTB crops and, therefore, have more valuable knowledge and expertise. However, more evidence is needed and surveys about adoptions must include clear gender and expertise disaggregation of trait preferences in order to provide more illuminating data.

Hypothesis 3 The predominance of informal seed systems has been a major contributor to slow uptake of MVs.

The response to this hypothesis depends on the crop and context. In some cases, the informal system has contributed to rapid uptake of MVs. For example, the C18 yam and *Shangi* potato variety originated through farmer selections, which spread through informal seed systems to achieve significant adoption. Both have subsequently been released through national programmes. Given the generally small amount of RTB crop seed made available through the formal system, most MVs that may originate in the formal system ultimately reach the farmer through an informal one. Additionally, there is also evidence that a failure to engage with informal seed systems may limit adoption: in Uganda there is a functioning informal seed system in drier areas which may have been partially undermined by a campaign‐style free delivery of sweetpotato vines.

However, the informal system has its limitations. One major flaw is that accurate or consistent names are not reliably shared along with varieties, often leading to multiple local names, so that mix‐ups are common. This problem may be more severe with cassava and sweetpotato, where planting material is shared as vines and cuttings, compared to yam and potatoes which rely on visually distinctive tubers as planting material. For example, the C18 yam variety has tubers which end in toe‐like protuberances, quite unlike other yam varieties. For cassava, varietal names and identification are often more challenging, especially where there are many cultivated varieties, and varieties that are similar in appearance. For example, in Nigeria, a variety described by farmers as ‘419’ in one location might appear to correspond to TME 419, a well‐known released landrace. In fact, these two varieties are completely different: the former is most likely TMS‐IBA950289, which somehow got misnamed. More generally, genetic fingerprinting of cassava during the CMS adoption survey in Nigeria revealed that many farmers who believed they were growing a landrace actually were not and vice versa. Mix‐ups can also happen with yam and potato in the informal system. For example, in Kenya in the 1980s, Desiree a Dutch variety was mixed up with a farmer selected ‘Desiree’, which looked similar but lacked quality, thus confounding many adoption decisions. Furthermore, formal seed systems can play a key role in sustaining a reliable supply of known varieties which are then more widely disseminated locally through the informal system. For example, a decision to replace varieties sold by a major certified potato seed producer in Kenya was reflected in subsequent adoption of that variety noted in the Meru case study (Ian Barker personal communication). Similarly, more intensive distribution of planting material of hybrid bananas in Tanzania appeared linked to greater adoption, although much uptake depended on the informal sector (Smale & Tushemereirwe, 2007).

## Future directions for breeding programmes, dissemination and adoption surveys

The importance of paying attention to demand in the form of consumer preferences is consistent with the emerging breeding paradigm of demand‐led breeding (https://www.syngentafoundation.org/demand‐led‐breeding‐0). This work will require well‐structured varietal product profiles that pay much closer attention to consumer preferences carefully disaggregated by region, gender and other social differences.

Despite some recent work, much more information is needed to understand and address gender differences in consumer preferences. This will entail broader use of methods by breeding programmes to ensure that gender differentiated information can be captured and used effectively. Research planned with the CGIAR Excellence in Breeding Platform (https://www.cgiar.org/cgiar‐excellence‐in‐breeding‐platform/) and the CGIAR Gender and Breeding Initiative (http://www.rtb.cgiar.org/gender‐breeding‐initiative/) is promising, but broader use is still needed. Building linkages from consumer preferences to trait identification and high throughput protocols for screening for quality traits are considered high priority and is also being pursued by the RTBFoods project (https://www.cirad.fr/en/news/all‐news‐items/press‐releases/2018/rtbfoods). Key traits that drive adoption need to be incorporated into the selection strategies used by breeders (Bechoff *et al*., 2018).

For some RTB crops, where breeding is especially challenging, and marked consumer preferences are known, more attention could be paid to selection and distribution of landraces, which have attained high adoption rates in some cases, particularly for yam.

Many of the most widely adopted MVs in RTB crops are farmer selections (e.g. *Shangi* in Kenya). This suggests that the final stages of selection in breeding programmes and the release processes are not working optimally and not responding to a large amount of perceived demand. The alternative is to have more inclusive and demand‐led priority setting for key traits, which engage actor networks more closely (Sanya *et al*., 2017). Farmers’ opinions and choices can guide dissemination through tools such as Tricot (van Etten *et al*., 2019), and thus enable better dissemination through the informal system rather than trying to work around it. Closer attention needs to be paid to ensuring trueness to type in the formal system and ensuring consistent varietal naming at the local level so that farmers actually know what they are getting https://www.rtb.cgiar.org/news/fostering‐a‐sustainable‐cassava‐seed‐system‐for‐nigeria/.

Adoption studies are regularly carried out to show the uptake of modern varieties. Most studies have estimated adoption rates of MVs without considering the specific traits which underpin adoption decisions or the typology of male and female farmers adopting these varieties. These should be modified to gather explicitly information about trait preferences and gender differences, which have not been well captured in previous surveys. This also need to consider that farmers often grow a portfolio of different varieties sometimes with contrasting preferences for the same trait, such as wanting both bitter and sweet varieties in the case of cassava.

Surveys where experts estimate the area under adoption of MVs (breeder elicitations) have often led to significant overestimations of adoption (e.g. sweetpotato in Uganda) and most likely underlies the high adoption figure reported for the C18 yam variety in Côte D’Ivoire in 2009. Previous farmer surveys to assess varietal adoption have had significant limitations. In some crops where there are more distinctive varietal differences such as tuber shape and colour in potato, farmer surveys may provide more consistent data, as was the case in Kenya. However, as farmers often misrepresent the identity of the varieties they are growing, using DNA fingerprinting to correctly identify the varieties being grown will be an essential addition to adoption studies, and should be made more feasible by the declining cost of these services.

## Author contribution

**Graham Thiele:** Conceptualization (lead); Investigation (lead); Methodology (lead); Writing‐original draft (lead); Writing‐review & editing (lead). **Dominique Dufour:** Investigation (supporting); Writing‐original draft (supporting); Writing‐review & editing (supporting). **Phlippe Vernier:** Investigation (supporting); Writing‐original draft (supporting). **Robert Mwanga:** Investigation (supporting); Writing‐original draft (supporting). **Monica Parker:** Investigation (supporting); Writing‐original draft (supporting). **Elmar Schulte‐Geldermann:** Investigation (supporting); Writing‐original draft (supporting). **Béla Teeken:** Investigation (supporting); Writing‐original draft (supporting). **Tesfamicheal Wossen:** Investigation (supporting); Writing‐original draft (supporting). **Elisabetta Gotor:** Investigation (supporting); Writing‐original draft (supporting). **Enoch Mutebi Kikulwe:** Investigation (supporting); Writing‐original draft (supporting). **Hale Tufan:** Investigation (supporting); Writing‐original draft (supporting). **Sophie Sinelle:** Investigation (equal). **Amani Michel Kouakou:** Investigation (equal). **Michael Friedmann:** Investigation (supporting); Writing‐original draft (supporting). **Vivian Polar:** Investigation (supporting); Writing‐original draft (supporting). **Clair H Hershey:** Investigation (supporting); Writing‐original draft (supporting); Writing‐review & editing (supporting).

### Peer Review

The peer review history for this article is available at https://publons.com/publon/10.1111/ijfs.14684.

## Data Availability

Data sharing is not applicable to this article as no new data were created or analysed in this study. Ethics approval was not required for this research as it was based on a review of already published documents. The authors’ have no conflicts of interest with the review content and findings.

## Notes

^1^ A varietal *product profile* is a description of the priority traits which breeders seek to include in varieties being developed. Other papers in the special issue use *product profile* or *food product profile* to refer to the characteristics that the derived food product should have.

^2^ The term *seed* is used in the context of RTB crops to refer to vegetative propagules rather than botanical seed.

^3^ This adoption share emanates primarily from the CGIAR ‘s work in RTB crops, with the exception of yam and banana.

^4^ Although, as the case study will make clear, the adoption area under C18 appears to be overestimated, biasing upward the MV adoption figures for yam.

^5^ When adoption is defined as the percentage of adopting farmers, the rate is 60%. However, in this paper, adoption is defined as area under MVs.

^6^ As population has increased in Nigeria and wild animals become rarer this preference may have diminished.

^7^ Preliminary molecular analysis of *Shangi* seems to place it in the CIP genebank germplasm. It may have originated from CIP germplasm distributed from Peru to Kenya, Burundi, and Tanzania in the 1990s (International Potato Center (1995). Program Report: 1993‐1994. Lima, CIP**:** 192 p.).

^8^
*Florido* is called “matches”, in Ghana, because it can be grown from a piece of tuber as small as a matchbox (Philippe Vernier personal communication).
